# Biological Prior Knowledge-Embedded Deep Neural Network for Plant Genomic Prediction

**DOI:** 10.3390/genes16040411

**Published:** 2025-03-31

**Authors:** Chonghang Ye, Kai Li, Weicheng Sun, Yiwei Jiang, Weihan Zhang, Ping Zhang, Yi-Juan Hu, Yuepeng Han, Li Li

**Affiliations:** 1 Agricultural Bioinformatics Key Laboratory of Hubei Province, College of Informatics, Huazhong Agricultural University, Wuhan 430070, China; chonghangye@webmail.hzau.edu.cn (C.Y.); kaili@webmail.hzau.edu.cn (K.L.); weichengsun@webmail.hzau.edu.cn (W.S.); yiweijiang@webmail.hzau.edu.cn (Y.J.); pingzhang@webmail.hzau.edu.cn (P.Z.); 2 Hubei Hongshan Laboratory, Wuhan 430070, China; whzhang@wbgcas.cn; 3 State Key Laboratory of Plant Diversity and Specialty Crops, Wuhan Botanical Garden, Chinese Academy of Sciences, Wuhan 430074, China; 4 School of Computer, BaoJi University of Arts and Sciences, Baoji 721016, China; 5 Department of Biostatistics, School of Public Health, Peking University, Beijing 100191, China; yijuanhu@bicmr.pku.edu.cn; 6 Beijing International Center for Mathematical Research, Peking University, Beijing 100871, China

**Keywords:** genomic prediction, AI breeding, latent genetic interaction, maize, soy

## Abstract

**Background/Objectives:** Genomic prediction is a powerful approach that predicts phenotypic traits from genotypic information, enabling the acceleration of trait improvement in plant breeding. Traditional genomic prediction methods have primarily relied on linear mixed models, such as Genomic Best Linear Unbiased Prediction (GBLUP), and conventional machine learning methods like Support Vector Regression (SVR). Traditional methods are limited in handling high-dimensional data and nonlinear relationships. Thus, deep learning methods have also been applied to genomic prediction in recent years. **Methods:** We proposed iADEP, Integrated Additive, Dominant, and Epistatic Prediction model based on deep learning. Specifically, single nucleotide polymorphism (SNP) data integrating latent genetic interactions and genome-wide association study results as biological prior knowledge are fused to an SNP embedding block, which is then input to a local encoder. The local encoder is fused with an omic-data-incorporated global decoder through a multi-head attention mechanism, followed by multilayer perceptrons. **Results**: Firstly, we demonstrated through experiments on four datasets that iADEP outperforms existing methods in genotype-to-phenotype prediction. Secondly, we validated the effectiveness of SNP embedding through ablation experiments. Third, we provided an available module for combining other omics data in iADEP and propose a novel method for fusing them. Fourthly, we explored the impact of feature selection on iADEP performance and conclude that utilizing the full set of SNPs generally provides optimal results. Finally, by altering the partition of training and testing sets, we investigated the differences between transductive learning and inductive learning. **Conclusions:** iADEP provides a new approach for AI breeding, a promising method that integrates biological prior knowledge and enables combination with other omics data.

## 1. Introduction

Genomic prediction (GP) was initially proposed as a new method of selection decision that leverages high-density genomic markers [1]. GP builds a prediction model using a training set consisting of known phenotypes and genotypes. With this model, individuals with known genotypes but unknown phenotypes can be predicted, enabling early individual selection based on genomic estimated phenotypic values. This approach reduces the cost of field phenotype identification, shortens breeding years, and improves genetic gain [2].

Traditional genomic prediction methods are primarily based on statistical models, which have been divided into two categories: the direct method and the indirect method [3]. The direct method treats individuals as random effects and constructs their genetic relationship matrix as the covariance matrix based on genetic information from the training and testing sets [4]. The phenotype values for the testing set are obtained by solving the mixed linear model, represented by methods such as genomic best linear unbiased prediction (GBLUP) [5]. The indirect method estimates markers’ effects on the training set [6]. The phenotype values of the testing set are obtained by accumulating the marker’s effects based on the training set. Common methods include BayesA, BayesB, BayesC, etc. [1,7]. However, the traditional GP methods face several challenges. Specifically, the direct method requires the genotype data of the testing set, necessitating the reconstruction of the GP model for new individuals. The indirect method relies on the prior distribution of the biological marker effect and its variance, which limits its ability to capture the genotype-phenotype relationship. Furthermore, both methods struggle to effectively capture nonlinear relationships or handle multi-dimensional and multi-omic data. To overcome these limitations, machine learning (ML) methods have been further developed [8]. Random forest (RF), ridge regression (RR), support vector regression (SVR), and other methods have yielded great prediction accuracy in breeding wheat for rust resistance [9]. A comparison of the prediction accuracy of different machine learning methods for the reproductive traits of Nellore cattle reveals that SVR is the most suitable method for these traits [10].

Deep learning (DL), a subfield of ML, is an advanced technique that excels in handling high-dimensional big data and analyzing involved interaction or relationship [11,12]. The deep convolutional neural network (DeepGS), the first DL model in GP proposed by Ma et al., is a model composed of convolutional neural networks (CNN) and uses wheat DArT (Diversity Array Technology) markers to predict phenotypes [13]. Similarly, based on CNN, deep neural network genomic prediction (DNNGP) takes high-dimensional SNPs (single nucleotide polymorphisms) markers through PCA (principal component analysis) as partial input and integrates multi-omic data to jointly predict phenotypes [14]. In contrast to the CNN architecture, GPformer is a modified model of the Encoder in Transformer, which can greatly expand the features extraction receptive field and adopts an attention mechanism to capture global information [3,15]. Furthermore, the integration of genome-wide association study (GWAS) results as prior knowledge can improve GPformer prediction performance.

So far, the markers (usually SNPs) are commonly encoded by 0, 1, and 2 to distinguish homozygous or heterozygous states in the aforementioned DL-based methods. However, this encoding approach lacks discrimination for different SNPs, which contradicts the inductive bias of local similarity in CNN. DNNGP allows for the application of a variety of omics data to predict. However, at the input layer, DNNGP only employs one data type, which does not fully utilize the information of multi-omic. Additionally, the auto-correlation attention mechanism used in GPformer is derived from the series periodicity, introducing an inductive bias inconsistent with biological markers although this mechanism effectively handles long-range sequences [16]. Moreover, the effects of genes themselves, including additive effects, dominant effects, and epistatic effects (interactions between genes), have been partially overlooked [17].

To address these issues, we proposed the Integrated Additive, Dominant, and Epistatic Prediction (iADEP) model, a deep learning-based model designed to improve genomic prediction performance by integrating biological prior knowledge and optional multi-omic data. Specifically, iADEP mainly consists of three parts: SNP embedding, local encoder-global decoder and multilayer perceptrons (MLP), involving ResNet [18] and multi-head attention mechanism [15]. In the SNP embedding block, SNP data are inputted and integrated with GWAS and Latent Interaction Testing (LIT) [19] as biological prior knowledge to generate SNP embedding. In the global decoder block, SNP data can be inputted and merged with the output of the local encoder block, and additional omics data can be inputted and merged with the SNP output. The prediction performance of iADEP was compared with other representative methods like GBLUP, RR, SVR, RF, XGBoost (eXtreme Gradient Boosting), and DNNGP from various models in diverse datasets. The results demonstrate that iADEP is a promising method that can successfully predict multiple traits in diverse datasets. Subsequently, we validated the effectiveness of SNP embedding as biological prior knowledge by ablation experiments. Furthermore, we evaluated the performance of incorporating other omics data into the global decoder block. Finally, we investigated the impact of feature selection on iADEP and explored the distinctions between transductive learning and inductive learning.

## 2. Materials and Methods

### 2.1. Dataset Used for Genomic Prediction

Five datasets in which the genetic markers are SNPs and have large sample sizes were selected in this study, including millet827, spruce1722, soy5014, maize5820, and springbarley2463. Detailed information on the SNP and traits for all datasets in provided in Table S1. Millet827, consisting of 827 foxtail millet cultivars collected from China, was used to explore genotype-dependent microbial effects [20]. Each line has a sequence of 161,562 SNPs and can be downloaded from CropGS-Hub [21]. The SNPs were filtered out by PLINK [22] with minor allele frequency (MAF) < 0.05 and genotype calling rate < 0.1, yielding a total of 75,780 SNPs. This dataset consists of twelve agronomic traits, including six growth traits and six yield traits. Among these traits, the hundred kernel weight (HKW) trait was excluded due to its extremely low heritability ($h_{snp}^{2}=0.0069$).

Spruce1722, derived from the SmartForests project team [23], consists of 1722 white spruce samples with a sequence of 6930 SNPs without missing data and three traits. The SNPs were filtered out by PLINK with MAF < 0.05, yielding a total of 5679 SNPs.

Soy5014 was obtained from the SoyNAM population, which contains recombinant lines (RILs) derived from 40 biparental populations [24]. Soy5014 consists of 5014 samples with a sequence of 4234 SNPs and three traits. Spruce1722 and Soy5014 datasets can be downloaded from the previous study [25].

Maize5820 is a maize NCII population of 5820 F_1_ hybrids created from the cross of 30 diverse elite paternal lines and maternal inbred lines which were a subset of the CUBIC population [26]. The SNPs called in all 224 inbred lines by whole-genome resequencing were filtered out by PLINK with MAF < 0.05 or expected missing rate > 10% and was pruned with an LD threshold of 0.3. The maize5820, containing the resulting 156,269 SNPs and 18 agronomic traits, can be downloaded from CropGS-Hub or a previous study [27].

Springbarley2463 was collected from 2628 plots of 565 spring barley lines from Nordic Seed A/S, including genotype, phenotype, and metabolome data, which are available in a public accessible repository [28]. Each line had 3889 SNP markers and five malting quality traits. In addition, 24,018 metabolomic features were obtained for each sample from nuclear magnetic resonance spectra for wort samples produced from each experimental plot. The detailed descriptions of these traits and metabolomic features are reported in Guo et al. [29]. Samples with missing phenotypic data were filtered out, and missing SNP data were imputed using mean imputation followed by rounding. Finally, spring barley dataset consists of 563 lines and 2463 samples. This dataset was only used to study the application of iADEP in predicting phenotypes incorporating other omics data.

### 2.2. Heritability Estimation

The heritability for each trait was estimated using an SNP-based method, denoted as $h_{snp}^{2}$ [30]. The genetics complex trait analysis (GCTA) was used to estimate the phenotypic variance explained by SNP through genome-based restricted maximum likelihood (GREML) method [31]. The estimated heritability provides a reference significance for the genomic prediction results.

### 2.3. iADEP Algorithm

iADEP consists of three parts: SNP embedding, local encoder-global decoder, and MLP regression. SNP embedding block distinguished different SNPs based on biological prior knowledge of GWAS and LIT. In the local encoder block, it performed dimensionality reduction and encoded locally fused information, while the global decoder block employed multi-head attention mechanism to extract the globally fused features. In the global decoder block, it is possible to add other omics data to fuse genomic and other omics features. Finally, using MLP as regressor, the predictive results were obtained. Each part is described as follows.

In classical quantitative genetic methods, the genotypic value can be decomposed into the sum of additive, dominant, and epistatic parts as follows [32]:

(1) G=A+D+I

where G is the genotypic value, A is the sum of the additive effects attributable to the separate loci, D is the sum of the dominance deviations, and I is the sum of interaction deviation or epistatic deviation.

Based on this hypothesis, we employed an embedding methodology to encode each SNP that contain these three parts. Specifically, GWAS based on a linear mixed model can capture the additive effects between SNPs and phenotypes. Each SNP encoded as heterozygous state may exhibit within-locus interactions, known as dominant effect. Additionally, the interactions between different SNPs can be captured using the LIT algorithm [19]. LIT uses a flexible kernel-based framework to test for latent genetic interactions in genome-wide association studies, yielding output formats that are similar to GWAS. Therefore, the representation of a SNP depends on which homozygous and whether heterozygous and its initial representation can be encoded as follows:

(2) $$\left\{\begin{array}{ll} \left[-\log GWAS\;p-value,\;0,-\log LIT\;p-value\right] & ,\;if\;SNP=1\\ \left[0,1,-\log LIT\;p-value\right] & ,\;if\;SNP=0\\ \left[-\left(-\log GWAS\;p-value\right),\;0,-\log LIT\;p-value\right] & ,\;if\;SNP=-1 \end{array}\right.$$

**Figure 1:**
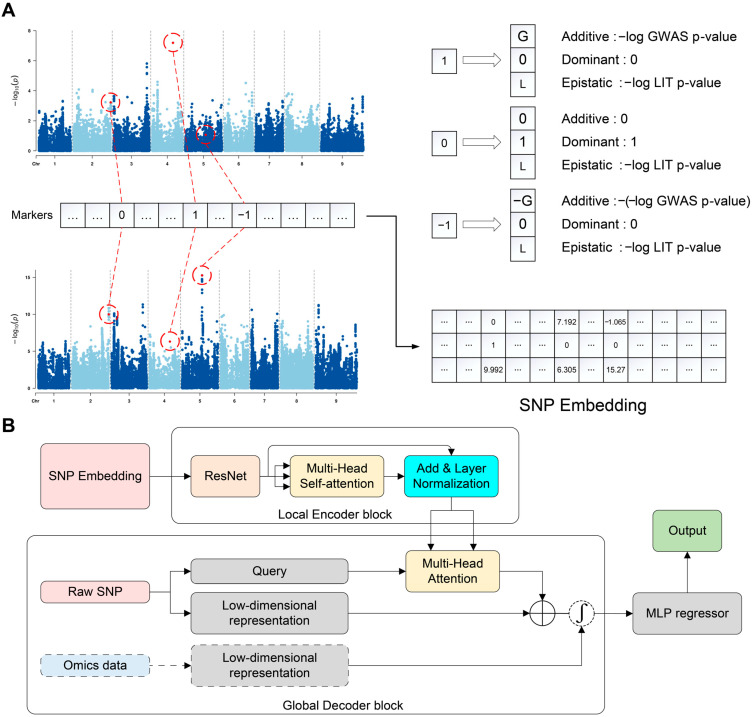
Overview of iADEP framework. (**A**) Illustration of SNP embedding using trait MSW as an example. The upper left panel shows the Manhattan plot of GWAS; the lower left panel shows the Manhattan plot of LIT. The right panel gives the result of SNP embedding. (**B**) The construction of iADEP, where dashed line-boxes indicate sections which are optional for other omics data. MSW, main stem width.

Figure 1A provides a detailed description of the process of SNP embedding, where GWAS can be performed in R package “rrBLUP” [6] or EMMAX (beta) [33] software, and LIT can be performed in R package “lit” [19].

Local encoder-global decoder mainly consists of a series of residue connections and a multi-head attention mechanism. The output of SNP embedding can be considered as making each SNP contain more information. In contrast to traditional CNN architectures employed in previous studies, ResNet block demonstrates advantages in preventing gradient explosion and vanishing, while concurrently preserving more low-level features [18]. Therefore, we extracted local features by ResNet block. The interaction information between local features is extracted through multi-head self-attention mechanism. Multi-head self-attention creates a high-level understanding of the data, including interaction among local features, as illustrated below:

(3) $$Attention\left(Q,\;K,\;V\right)=softmax\left(\frac{QK^{T}}{\sqrt{d_{k}}}\right)V$$

where Q, K, and V are query, key, and value, respectively, and $d_{k}$ is the dimension of K. Q, K, and V are the same vectors in self-attention [15]. Eventually, a high-level feature containing local information and interaction information is obtained in the local encoder block.

Residue connection and multi-head attention mechanism are also employed in the global decoder block. First, we applied two-layer linear mappings with LeakyReLU as the activation function to the raw SNP, which helps simplify the global information to create a low-dimensional representation. This low-dimensional representation with the output of local encoder block is integrated through a multi-head attention layer to fuse local and global information. Additionally, residual connections were employed to supplement the fused features with global information (Figure 1B). The LeakyReLU activation function is as follows [34]:

(4) $$y_{i}=\left\{\begin{array}{c} x_{i},\;if\;x\geq 0\\ \frac{x_{i}}{a_{i}},if\;x<0 \end{array}\right.$$

where i represents the index of the input or output, and $a_{i}$ is a fixed parameter, defaulting to 100.

If other omics data are available, the global decoder block provides a module for combining them. Taking metabolomic features as an example, similar to raw SNP data, a two-layer linear mapping is used to obtain a low-dimensional representation. This low-dimensional representation is then combined with the output of the global decoder for the SNP data using the following feature fusion method:

(5) $$\begin{array}{cc} Fusion & \left({Output}_{SNP},\;{Output}_{met}\right)\\ = & {Output}_{met}+Sigmoid\left(Max\left({Output}_{SNP},\;{Output}_{met}\right)\right) \end{array}$$

where the ${Output}_{SNP}$ is the output of the global decoder for the SNP data and ${Output}_{meta}$ is the low-dimensional representation. The reason for employing such fusion approach is that, according to the central dogma, the features of the metabolome manifest later than genome and are closer to phenotypic traits. Therefore, the features of the metabolome not only exhibit intersections with features on the genome, but also contain more hidden phenotypic information. To capture these metabolic features more effectively, we used non-linear function max and activation function sigmoid to avoid redundancy in the features. Additionally, residue connection by adding the metabolomic feature to capture more metabolomic information.

The final features were used to predict the phenotype value through a three-layer MLP that incorporates LeakyReLU activation function and dropout layers. We chose mean squared error (MSE) as the initial loss function and used L2 regularization to adjust the training process to reduce generalization errors. Figure 1 shows the entire architecture of iADEP.

### 2.4. Model Construction of Existing Representative Methods

To assess the effectiveness of iADEP more objectively, we selected representative methods of different types on the same dataset. Specially, we selected the classical method GBLUP based on linear mixed model, the linear regression method ridge regression, random forest as the representative method of tree models, SVR as the classical ML method, XGBoost as the ensemble learning method, and the latest deep method DNNGP. Here is a brief introduction to these methods.

GBLUP, genomic best linear unbiased prediction, is a linear mixed model derived from BLUP. It replaces the pedigree relationship matrix with a genomic relationship matrix [5], which is formulated as follows:

(6) $$G=\frac{ZZ^{T}}{2\sum p_{j}\left(1-p_{j}\right)}$$

where Z represents the standardized genotype matrix, and $p_{j}$ denotes the allele frequency of locus j. GBLUP can be implemented using the R package “rrBLUP” [6].

RR, ridge regression, introduces a regularization term to the ordinary least squares regression to improve the stability and generalization ability of the model [35]. This regularization term penalizes the sum of squared coefficients. The formula for RR is as follows:

(7) $$y=X\hat{\beta }+\lambda \sum_{j=1}^{p}\beta_{j}^{2}$$

where y is the predicted response variable, X is the design matrix of predictors, $\hat{\beta }$ is the vector of estimated regression coefficients, λ is the ridge parameter, j is an index used to refer to the j-th coefficient in the regression coefficient vector $\hat{\beta }$, $\beta_{j}$ is the coefficients of the predictors, and p is the number of predictors.

RF, random forest, is an ensemble tree model that combines multiple decision trees to make predictions [36]. It achieves diversity by training each decision tree on a random subset of data and features. The final prediction is obtained by voting or averaging the predictions of individual tress.

SVR, support vector regression, is a non-linear regression model based on support vector machines [37]. It maps the training sample to a high-dimensional feature space and constructs an optimal hyperplane for regression prediction. SVR establishes a boundary by finding support vectors to minimize the error between predicted values and observed values. If linearity is not possible in the high-dimensional space, it can introduce the relaxation variables $\varepsilon_{i}\;\geq 0$. The formula is as follows:

(8) $$\left\{\begin{array}{l} \underset{w,b}{\min}\frac{1}{2}w^{T}w+C\sum_{i=1}^{N}\varepsilon_{i}\\ s.t.\;y_{i}\;(w^{T}x_{i}+b)\geq 1-\varepsilon_{i},\;\varepsilon_{i}\geq 0 \end{array}\right.$$

where w is the weight vector, b is the intercept, C is the regularization constant, N is the sample size, i is the index of the sample, $\varepsilon_{i}$ is the relaxation variables, and $y_{i}$ and $x_{i}$ are paired training set features and labels. RR, RF, and SVM can be implemented in Python3.10.9 using “scikit-learn1.2.0” [38].

XGBoost, eXtreme Gradient Boosting, is based on the gradient boosting framework and combines multiple weak learners to create a strong ensemble model [39]. XGboost can be implemented in Python using “xgboost2.0.0”.

DNNGP, deep neural network genomic prediction, is based on deep CNN [14]. It contains one input layer, three convolutional layers, one batch normalization layer, two dropout layers, one flattening layer, one dense layer, and one output layer. The core block convolutional layer is presented as follows [40]:

(9) $$s\left(t\right)=\left(f*k\right)\left(t\right)=\sum_{x}k\left(t-x\right)f\left(x\right)$$

where k represents the kernel size, convolution is the transformation of f into s(t). In DNNGP, the kernel size is set to 4, which is much smaller than the number of SNPs. To address this, PCA is applied to the SNP data, and the top 95% of feature variance is extracted. We have also adopted this approach when using DNNGP. On springbarley2463 dataset, the CNN kernel size is much smaller than the length of the metabolomic features (24,018). Thus, we applied PCA to the metabolomic features, selecting the top three PCs that accounted for 99% of the feature variance. These three features were then concatenated with the SNP data.

### 2.5. Model Evaluation

In this study, we implemented a 5-fold cross-validation scheme for a more rigorous evaluation of different methods. Each dataset was randomly divided into five folds, with four folds used as the training set and the remaining fold as the testing set. To make sure that phenotype data were not leaked owing to the fact that the *p*-values of GWAS and LIT are functions of the phenotype, GWAS and LIT were only performed on the training set, where only the springbarley2463 dataset is based the averaged values of different plots on the same line. We used Pearson correlation coefficient (PCC) as an evaluation metric and there are two schemes to evaluate prediction performance. The first scheme involves using PCC between the observed phenotypes and corresponding predicted values on each test fold. The average and standard error of the PCC are used as performance metrics. The second scheme involves recording the predicted values for all test sets and calculating a single PCC across all observed phenotypes. In this study, only the second scheme is used to investigate the differences between transductive learning and inductive learning, while the first scheme is used for all other experiments. The formula for PCC is as follows:

(10) $$PCC=\frac{Cov\left(X,Y\right)}{\sigma_{X}\sigma_{Y}}$$

where $Cov\left(X,Y\right)$ is the covariance between variable X and Y, $\sigma_{X}$ is the standard deviation of variable X, $\sigma_{Y}$ is the standard deviation of variable Y, X and Y correspond to phenotype observations and predictions, respectively.

## 3. Results

### 3.1. Overview of iADEP Architecture

iADEP consists of three parts: SNP embedding, local encoder-global decoder, and MLP regression (Figure 1). SNP embedding block distinguishes different SNPs based on biological prior knowledge of GWAS and LIT (Figure 1A). The local encoder block contains ResNet, multi-head self-attention layer and layer normalization, which are used to perform dimensionality reduction and encode locally fused information. The global decoder block employs a multi-head attention mechanism to extract the globally fused features and residue connection to retain partial global information. Additionally, the global decoder block provides a module that allows the addition of other omics data to fuse genomic and other omics features. Finally, the prediction results are obtained by an MLP as a regressor (Figure 1B).

The prediction performance is evaluated using the PCC between the predicted values and the observed values. The overall prediction performance of iADEP is positively correlated with heritability (Figure S1). SNP-based heritability ($h_{snp}^{2}$), which is smaller than $h^{2}$, was calculated to provide a reference for GP [41,42]. The heritability differences between different species may be due to differences in planting environments, which will not be discussed here. In general, iADEP exhibits higher prediction performance for traits with higher $h_{snp}^{2}$. Specially, the days to anther (DTA) trait ($h_{snp}^{2}=0.8263$) in maize5820 dataset achieves the highest prediction performance of 0.940, whereas the diameter at breast height (DBH) trait ($h_{snp}^{2}=0.1674$) in spruce1722 dataset has the lowest prediction performance of 0.286.

### 3.2. Prediction Performance of iADEP and Comparison with Other Methods

**Figure 2:**
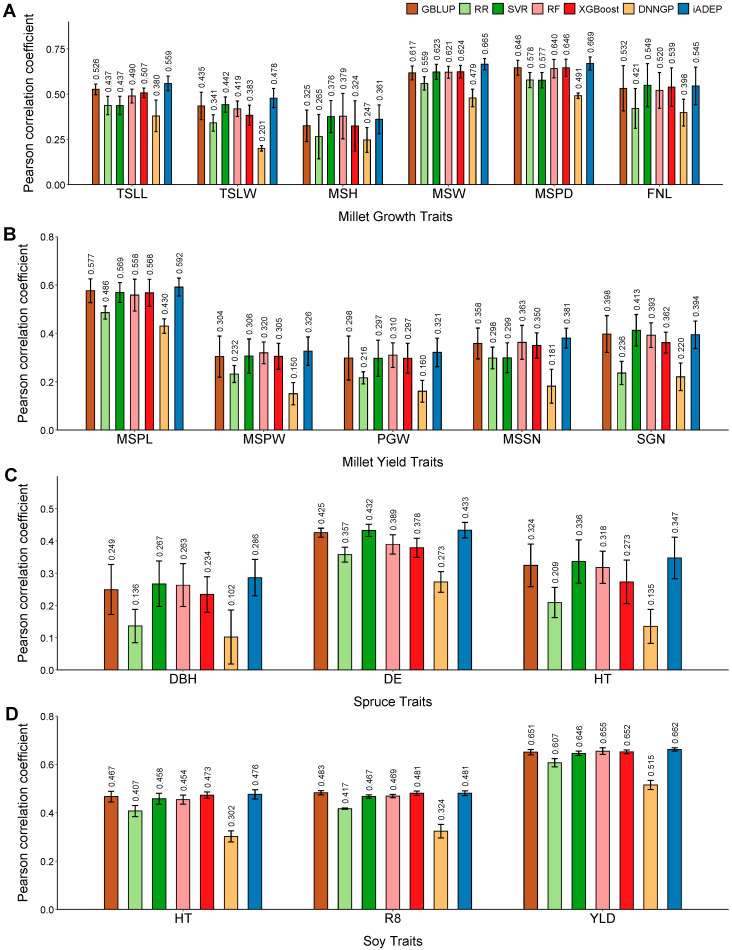
Prediction performance comparison of seven methods on three datasets: millet827, spruce1722 and soy5014. (**A**,**B**) Prediction performance on millet827 growth traits (**A**) and yield traits (**B**). (**C**) Prediction performance on spruce1722 dataset. (**D**) Prediction performance on soy5014 dataset. TSLL, top second leaf length; TSLW, top second leaf width; MSH, main stem height; MSW, main stem width; MSPD, panicle diameter of the main stem; FNL, fringe neck length; MSPL, panicle length of the main stem; MSPW, main stem panicle weight; PGW, per plant grain weight; MSSN, spikelet number of the main stem; SGN, grain number per spike; DBH, diameter at breast height; DE, wood density; HT, height; R8, time to R8 developmental stage; YLD, yield.

We compared the prediction accuracy for GBLUP, RR, SVR, RF, XGBoost, DNNGP and iADEP on four datasets: millet827, spruce 1722, soy5014 and maize5820. Firstly, we compared all methods on the millet827 dataset, which is a relatively small dataset, containing six growth traits and five yield traits. In terms of growth traits, iADEP demonstrated the best overall prediction performance, outperforming all other methods except for the main stem height (MSH) trait and fringe neck length (FNL), where their performance was lower than that of RF and SVR. GBLUP and RF were found to be suboptimal methods. However, for example, the performance of GBLUP and RF was 4.8% and 4.4% lower than iADEP in main stem width (MSW) trait, respectively (Figure 2A). In terms of yield trait, iADEP also exhibited the superior comprehensive prediction performance across all traits, with the exception of the grain number per spike (SGN) trait, where it exhibited a slightly lower performance compared to GBLUP (0.4%) (Figure 2B).

We then tested and determined the prediction performance of all methods on another relatively small dataset, spruce1722, which contains three traits. Across the three traits, iADEP consistently achieved the highest prediction performance, followed by SVR and GBLUP (Figure 2C). Compared to SVR, iADEP showed the highest improvement in the diameter at breast height (DBH) trait, with an increase of 1.9%. The next highest improvement was observed in the height (HT) trait, with an increase of 1.1%, while there was almost no improvement in the wood density (DE) trait (0.1%).

The third dataset, soy5014, is a relatively large dataset that includes three traits. In terms of the height (HT) and yield (YLD) traits, iADEP demonstrates superior predictive performance compared to the second-best methods, XGBoost and RF, with improvements of 0.3% and 0.7%, respectively. However, for the time to R8 developmental stage (R8) trait, iADEP is slightly lower than GBLUP by 0.2% (Figure 2D). Overall, on the soy5014 dataset, iADEP exhibited the best overall performance, although its advantage over smaller dataset is not as significant.

**Figure 3:**
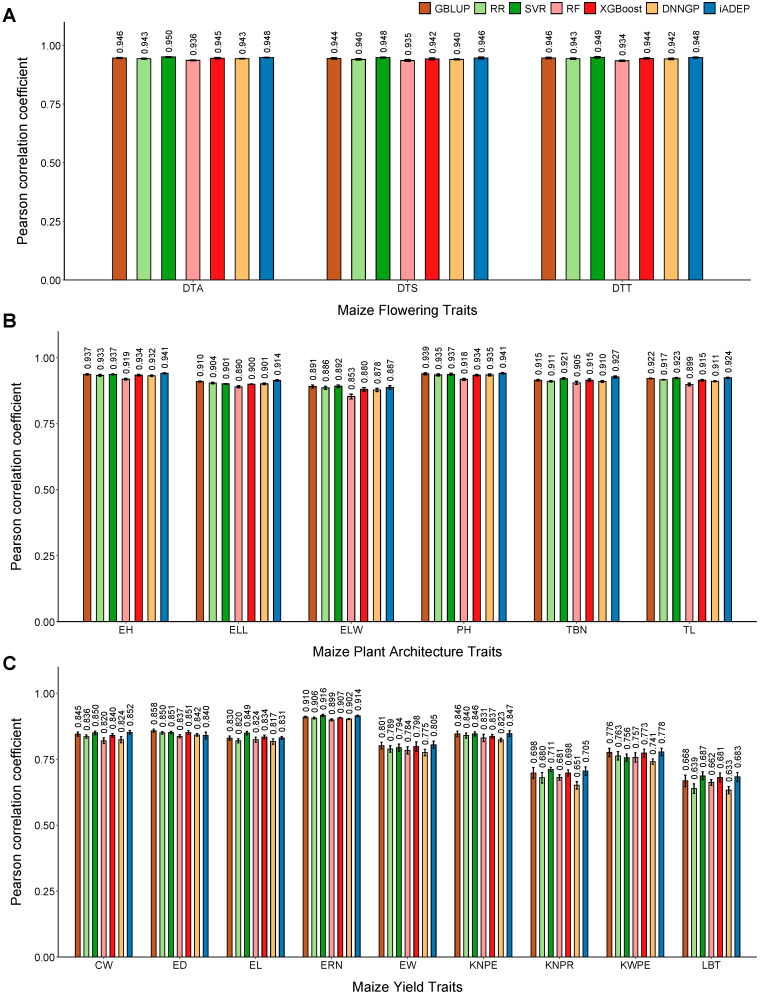
Prediction performance of seven methods on maize5820 dataset. (**A**–**C**) Prediction performance on maize5820 flowering traits (**A**), plant architecture traits (**B**), and yield traits (**C**). DTA, days to anther; DTS, days to silk; DTT, days to tassel; EH, ear height; ELL, ear leaf length; ELW, ear leaf width; PH, plant height; TBN, tassel branch number; TL, tassel length; CW, cob weight; ED, ear diameter; EL, ear length; ERN, ear row number; EW, ear weight; KNPE, kernel number per ear; KNPR, kernel number per row; KWPE, kernel weight per ear; LBT, length of barren tip.

The final dataset, maize5820, is the largest dataset containing the most samples, SNPs and traits. It includes 18 traits that can be categorized into three groups: flowering traits, plant architecture traits and yield traits. In terms of plant architecture traits, iADEP showed the best prediction performance except for the ear leaf width (ELW) trait, followed by SVR. For flowering traits, SVR exhibited the highest prediction performance, followed by iADEP. As for yield traits, iADEP, SVR and GBLUP all demonstrated satisfactory performance (Figure 3). Together, for 9 out of 18 traits, iADEP achieves the best performance, although the differences among the methods are minimal, which could be attributed to the high heritability of each trait on this dataset, indicating their inherent predictability.

In summary, for most of the traits tested, iADEP performs better than other methods with small to moderate magnitudes. The statistical analysis results are shown in Figure S5.

### 3.3. Ablation Experiments of SNP Embedding

To validate the effectiveness of biological prior knowledge, we conducted ablation experiments focusing on SNP embedding. Two conventional approaches were designed for this purpose (Figure S2):

Approach 1: one-hot encoding was employed, whereby the three possible states of SNPs, namely [1, 0, −1], were encoded as [[1, 0, 0], [0,1, 0], [0, 0, 1]], respectively.

Approach 2: Non-encoding was employed, but an additional channel was introduced to incorporate the SNPs information into the ResNet architecture. In this case, the states [1, 0, −1] were encoded as [[1], [0], [−1]], which is similar to methods such as DNNGP.

Ablation experiments were performed on four datasets to assess the effectiveness of SNP embedding (Table 1). Firstly, validation was conducted on millet827 dataset. In terms of growth traits, both the one-hot encoding and the non-encoding approaches resulted in a decrease in the prediction performance of iADEP. The most significant decrease was observed in the main stem width (MSW) trait, with a decrease of 2.7% and 3.2% for the two encoding approaches, respectively. As for the yield traits, only the main stem panicle weight (MSPW) trait showed a 1.2% improvement when employing one-hot encoding. However, for the other traits, both encoding methods led to a decrease in prediction performance.

On the spruce1722 dataset, SNP embedding consistently showed the highest prediction performance across the three traits, while one-hot encoding and non-encoding resulted in performance declines. Overall, one-hot encoding led to a decrease of 1% to 2%, while non-encoding resulted in a decrease of 2% to 6% in performance.

On the soy5014 dataset, SNP embedding also exhibits the highest prediction performance. Specifically, for the HT and R8 traits, both one-hot encoding and non-encoding result in significant performance declines, ranging from 1.8% to 4.4%. However, for the YLD trait, the differences among the three methods are minimal, with only a slight decrease of 0.3% observed.

On the maize5820 dataset, both one-hot encoding and non-encoding led to a decrease in prediction performance, and the results showed a larger standard deviation comprehensively. For flowering traits, this decrease is relatively small, ranging from 0 to 1%. However, for plant architecture traits, the decrease is more pronounced, except for the ear leaf width (ELW) trait, where the decrease is minimal. Other plant architecture traits show a decrease of 1% to 3%. As for yield traits, except for the kernel number per ear (KNPE) trait, which shows a slight decrease, the decreasing trend is more significant for other yield traits.

### 3.4. Genomic Prediction Incorporating Other Omics Data

In the global decoder block, we designed a module and feature fusion approach for other omics data (Figure 1B). In existing methods, such as DNNGP, multi-omic data including SNP are treated as markers without differentiation. Traditional machine learning methods primarily handle unidimensional data rather than multi-dimensional data. Therefore, for both traditional machine learning methods and DNNGP, we employed a multi-omic data integration strategy through feature concatenation. In contrast, the GBLUP method is incompatible with multi-omic data as it requires the construction of a genomic relationship matrix, which cannot be directly applied to multi-omic data. We compared the prediction performance of iADEP and other methods when combining other omics data on the springbarley2463 dataset, which contains SNP, metabolomic and phenotypic data.

**Figure 4:**
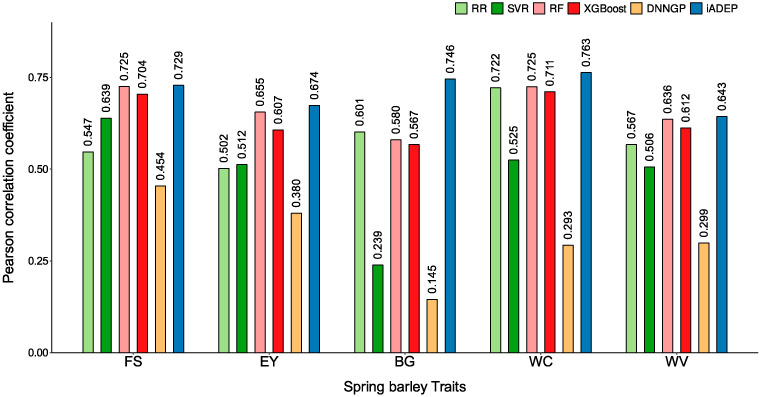
Prediction performance comparison of six methods on springbarley2463 dataset when incorporating other omics data. FS, filtering speed; EY, extract yield; WC, wort color; BG, beta glucan; WV, wort viscosity.

On the Springbarley2463 dataset, iADEP achieved the highest prediction performance across all traits tested, followed by RF (Figure 4). The statistical analysis results are shown in Figure S6. Particularly, iADEP exhibited a significant advantage in predicting the beta glucan (BG) trait, surpassing RR by 14.4%. It also performed well in predicting the wort color (WC) and extract yield (EY) traits. For the wort viscosity (WV) and filtering speed (FS) traits, although the improvement of iADEP was not as pronounced, it still outperformed RF by 0.75% and 0.33%, respectively. Together, iADEP outperforms other state-of-the-art methods when conducting genome prediction based on multi-omic data.

### 3.5. Effects of Feature Selection on iADEP

In previous studies, researchers have explored the potential of feature selection to improve the performance of genomic prediction. In terms of feature selection, these studies can be categorized into two approaches. The first approach randomly selects a certain percentage of markers, such as selecting randomly 20% of the markers [14,43]. The second approach considers GWAS-associated markers, such as selecting the top-k GWAS-associated markers [25,44]. In this study, we simultaneously considered markers associated with GWAS or LIT. We extracted the top 20%, 10%, and 5% SNPs associated with GWAS or LIT due to the variations in the number of SNPs across different datasets. Then, the union of these SNPs was taken to explore the impact of feature selection on prediction performance.

**Figure 5:**
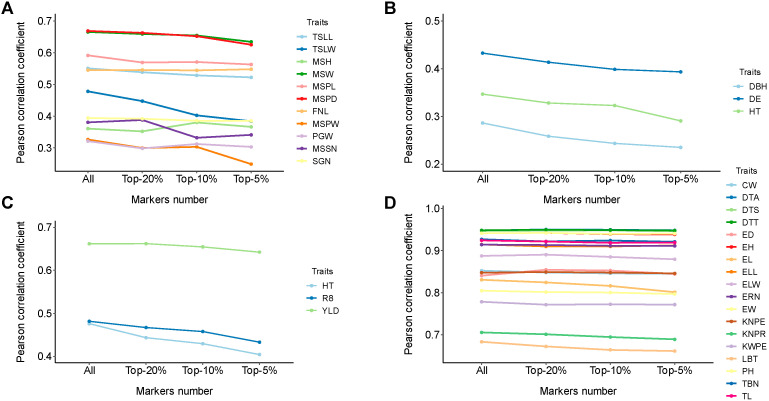
The changes of prediction performance with feature selection using thresholds of top-20%, top-10%, and top-5% SNPs for four datasets. (**A**–**D**) The prediction performance of feature selection under top-20%, top-10%, and top-5% thresholds for SNP selection on four datasets: millet827 (**A**), spruce1722 (**B**), soy5014 (**C**), and maize5820 (**D**). TSLL, top second leaf length; TSLW, top second leaf width; MSH, main stem height; MSW, main stem width; MSPD, panicle diameter of the main stem; FNL, fringe neck length; MSPL, panicle length of the main stem; MSPW, main stem panicle weight; PGW, per plant grain weight; MSSN, spikelet number of the main stem; SGN, grain number per spike; DBH, diameter at breast height; DE, wood density; HT, height; R8, time to R8 developmental stage; YLD, yield; DTA, days to anther; DTS, days to silk; DTT, days to tassel; EH, ear height; ELL, ear leaf length; ELW, ear leaf width; PH, plant height; TBN, tassel branch number; TL, tassel length; CW, cob weight; ED, ear diameter; EL, ear length; ERN, ear row number; EW, ear weight; KNPE, kernel number per ear; KNPR, kernel number per row; KWPE, kernel weight per ear; LBT, length of barren tip.

On the millet827 dataset, the impact of feature selection on prediction results can be roughly categorized into three types (Figure 5A). The first type is characterized by a positive correlation between model performance and the number of markers used. These traits included TSLL, TSLW, MSW, MSPD, and SGD trait, with TSLW showing the strongest trend (Figure 5A). The second type exhibits minimal changes with the variation in the number of markers (change less than 0.5%), such as the FNL trait. The third type presents a potential improvement in prediction performance as the number of markers decreased, although these improvements were generally small. For example, the MSSN trait showed a slight improvement, while only the MSH trait exhibited a significant increase. Overall, when the number of markers decreased to top 5–10%, except for the MSH trait, the performance tended to decline (Figure 5A).

On the spruce1722 and soy5014 datasets, all six traits exhibited a declining trend. Among them, the YLD trait in soy5014 dataset showed a slower decline, while the remaining traits displayed a sharper trend (Figure 5B,C).

On the maize5820 dataset, the overall prediction performance exhibited minimal changes with the variation in the number of markers, for example, all flowering traits showed a variation of less than 0.2%. Among other traits, the LBT and KNPR traits demonstrated a declining trend, while for the remaining traits, there might be a slight improvement in performance as the number of markers decreased, as observed in the case of the ED trait (Figure 5D). The results of all datasets are recorded in Table S4.

Together, for three out of the four datasets, model performance improves with the increase in SNPs used, which might be attributed to the number of markers in the dataset and SNP embedding. For maize5820 dataset, it shows no significant correlation between model performance and the number of SNP used. Out of the 35 traits examined, 23 traits show optimal prediction performance when all SNPs are used, 3 traits give optimal performance when the top 20% or top 10% SNPs are used, and the other 9 traits show minimal performance changes upon feature selection. Hence, in the majority of cases, selection of SNP does not improve prediction performance. Instead, iADEP tends to perform best when all markers are inputted as a whole.

### 3.6. Impact of Transductive Learning and Inductive Learning on Genomic Prediction

Transductive learning and inductive learning are terms used in ML, and they are frequently discussed in graph neural networks. In transductive learning, the models predict the labels of the training samples it has already seen, while the models of inductive learning predict the labels of unknown samples [45]. In traditional methods, GBLUP needs to construct a genetic relationship matrix based on the training and testing set when building the model, making it similar to transductive learning. However, iADEP uses a trained model to predict labels for unknown samples, thus falling in the paradigm of inductive learning.

**Figure 6:**
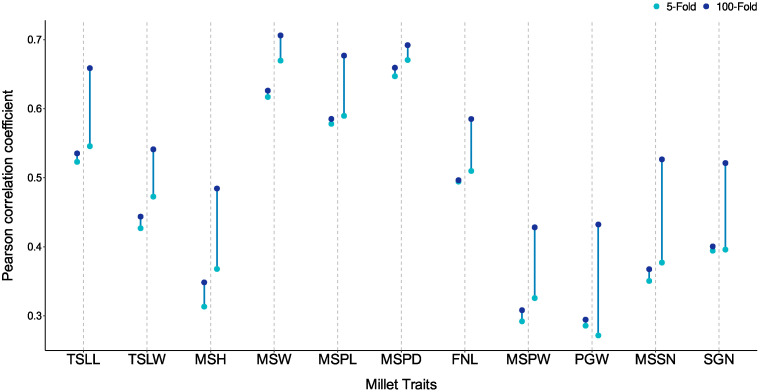
Comparison of prediction performance between 5-fold and 100-fold cross-validation for GBLUP and iADEP. For each trait, performance of GBLUP is represented on the left side of the dashed line, while performance of iADEP is represented on the right side. TSLL, top second leaf length; TSLW, top second leaf width; MSH, main stem height; MSW, main stem width; MSPD, panicle diameter of the main stem; FNL, fringe neck length; MSPL, panicle length of the main stem; MSPW, main stem panicle weight; PGW, per plant grain weight; MSSN, spikelet number of the main stem; SGN, grain number per spike.

To explore the differences between these two learning paradigms, we conducted 100-fold cross-validation on the millet827 dataset (Figure S3). In this setup, GBLUP still sees and uses all the samples, while iADEP has seen 99% of the samples, almost reaching a uniform level. We collected the predicted values for all test sets and calculated the prediction performance across the entire dataset. Figure 6 illustrates the changes in performance for both methods. Compared to the five-fold cross-validation, GBLUP showed only a modest improvement of around 1% for all traits, whereas iADEP exhibited significant improvement. This indicates that GBLUP is insensitive to the training and testing split of the samples since it needs to see all the samples, while the performance of iADEP is influenced by the partition of the training set.

## 4. Discussion

### 4.1. The Potential Role of SNP Embedding

With the recent breakthroughs in artificial intelligence, researchers are applying deep learning methods to build GP models. Currently, deep learning methods used for GP primarily rely on CNN architectures. For example, Zingaretti et al. built a CNN-based deep learning model that outperformed traditional linear statistical models in predicting traits with epistatic variances in allopolyploid strawberry species and blueberries [46]. Wang et al. built a DNNGP model containing three CNN layers, one batch normalization layer, and two dropout layers to predict quantitative traits in plants [14]. These CNN-based GP models have a common limitation. CNN models are based on two inductive biases: locality and translation equivariance [47], which are not compatible with the nature of biological markers, especially SNP (Figure S4). To address this issue, we introduced SNP embedding, an encoding strategy designed to be more consistent with biological logic.

Specifically, we proposed SNP embedding that integrates GWAS and LIT (Figure 1A). The primary potential role of SNP embedding is to differentiate SNPs at different loci through the statistical methods of GWAS and LIT. Therefore, in ablation experiments, the design of one-hot encoding and non-encoding approaches (both of which fail to differentiate SNPs) led to a decline in the prediction performance of nearly all traits compared to SNP embedding (Table 1 and Figure S2). Another potential effect of SNP embedding is to serve as a biologically meaningful form of pre-training, guiding the model toward a more accurate learning direction. In the feature selection experiments, we found that only a few traits exhibited improved prediction performance through feature selection in iADEP. This may be attributed to the fact that before model training, we have already marked different SNPs by SNP embedding. For instance, some uncorrelated SNPs may have initial embeddings close to [0, 0, 0], and their influence during the forward propagation of deep learning may be minimal, but not entirely disregarded.

### 4.2. Scalability of Multi-Omic Data Fusion

In the local encoder-global decoder block, we employed two rounds of multi-head attention mechanisms to extract local and global interaction information. In the global decoder block, we provided an available module for combining other omics data. We compared iADEP with other methods on the springbarley2463 dataset, excluding GBLUP due to its incompatibility with other omics data. Following the central dogma, we designed a method to fuse SNP features and metabolomic features. In this study, we only tested the combination of metabolomic data, but this block can be extended to combine additional information beyond omics data. For instance, in the future when more environmental data is collected, we could supplement the model with environmental data and design various fusion methods for combining genotypic and environmental data. This aligns with the purpose of the integrated genomic-enviromic prediction (iGEP) model proposed by Xu et al. [48].

### 4.3. Differences Between Transductive Learning and Inductive Learning

We explored the differences between transductive learning and inductive learning in GP. GBLUP is more like transductive learning as it requires accessing both training and testing samples to create a genomic relationship matrix. It is insensitive to the partitioning between training and testing data, making it a stable method and one of the most commonly used approaches in animal and plant breeding. However, its drawback is that for a new sample, it has to rebuild the model and make new prediction because the genomic relationship matrix needs to be reconstructed. On the other hand, machine learning and deep learning methods like iADEP are examples of inductive learning. They learn from the training set and apply that knowledge to unknown samples. Therefore, they are sensitive to the training sample size. Once a well-trained model is built, it can be applied to other scenarios, making it more flexible in practical applications. Moreover, the inductive learning framework of deep learning provides a strong foundation for future development, particularly in interpretability, as it excels at summarizing and generalizing information. Many biologists prefer to explore the mechanisms at the genetic level, while deep learning methods appear more like black box models. However, with the development of interpretable methods in deep learning, such as Shapley Value [49], Gradient-weighted Class Activation Mapping (GradCAM) [50], it is possible to identify the features that models considered most significant, thereby providing new biological understanding and novel research direction.

### 4.4. Limitations of iADEP

Although deep learning methods like iADEP have numerous advantages in terms of prediction performance and application scenarios, they also come with several limitations. Firstly, the model-building process is less flexible and relies on the empirical setting of hyperparameters. In iADEP, specific hyperparameters include learning rate, training epochs, dropout rate, activation functions, the head number of multi-head attention mechanism, and in the case of ResNet, the kernel size of the first residual block and the number of residual blocks. These settings are primarily adjusted based on the number of SNPs in the dataset and the range of phenotypic values for different traits. For example, millet827 dataset with a larger number of SNPs may require larger kernel sizes and more residual blocks to reduce to length of SNPs to reduce the dimension of SNPs. Thus, the training process is complex and involves more human intervention although a well-trained model is flexible in applications. In comparison, methods like GBLUP and SVR have simpler model-building processes and require fewer parameter settings. The hyperparameters of these methods are shown in Tables S2 and S3. Secondly, iADEP is sensitive to the sample size of the training data. Having a larger number of training samples often leads to better prediction results, as evident from the experiments with 100-fold cross-validation. Therefore, iADEP has higher requirements for training sample size, which might limit its application in practical breeding scenarios. However, the data-demanding feature of iADEP will profit from and benefit from this era of big data, especially the collection and organization of big data and large projects for crop breeding. Thirdly, deep learning methods need higher hardware requirements. In this study, for millet827, spruce1722 and soy5014 datasets, which have smaller sample sizes or SNP numbers, we conducted training and testing on CPUs. For maize5820, we performed training and testing on an A100 GPU. Compared to GBLUP, iADEP is significantly more time-consuming.

## 5. Conclusions

In summary, we built a deep learning model, iADEP, which effectively integrates biological prior knowledge and allows for the incorporation of other omics data. Our results show the prediction performance of iADEP to be promising compared to existing methods on four datasets from various plant species. Through ablation experiments, we validated the effectiveness of SNP embedding. Moreover, we provided a module to combine information from other omics data, even environmental data. Additionally, we investigated the impact of feature selection on iADEP and found that for the majority of the traits tested, model performance is better when using full SNP sites. Finally, we explored the differences between transductive learning and inductive learning in GP. We found that increasing the size of the training set significantly improved the performance of iADEP. This behavior indicates that large datasets coming out in the future will empower such deep learning-based approaches. In the era with great advances of AI, the insights gained by developing, evaluating, and applying iADEP in this study would contribute to the future widespread application of deep learning in plant breeding. Genomic prediction can facilitate the selection of superior plant varieties in contemporary breeding, thereby enhancing production efficiency and agricultural yield. Furthermore, as environmental data continue to be collected in the future, it may enable the identification of varieties better suited to withstand environmental impacts, further increasing productivity [51]. Notably, with the development of interpretability in the future, genome prediction based on deep learning may reveal more genetic-related mechanisms.

## Figures and Tables

**Table 1:** Results of ablation experiments on four datasets: millet827, spruce1722, soy5014, and maize5820.

Dataset	Trait	SNP Embedding	One-Hot Encoding	Non-Encoding
millet827	TSLL	**0.559 ± 0.040**	0.535 ± 0.033	0.533 ± 0.024
	TSLW	**0.478 ± 0.053**	0.438 ± 0.069	0.457 ± 0.048
	MSH	**0.361 ± 0.080**	0.348 ± 0.079	0.354 ± 0.089
	MSW	**0.666 ± 0.031**	0.639 ± 0.038	0.634 ± 0.042
	MSPD	**0.669 ± 0.036**	0.657 ± 0.030	0.649 ± 0.040
	FNL	**0.545 ± 0.104**	0.537 ± 0.112	0.544 ± 0.111
	MSPL	**0.592 ± 0.037**	0.582 ± 0.050	0.587 ± 0.039
	MSPW	0.326 ± 0.059	**0.338 ± 0.062**	0.291 ± 0.085
	PGW	**0.321 ± 0.059**	0.299 ± 0.061	0.281 ± 0.097
	MSSN	**0.381 ± 0.041**	0.351 ± 0.040	0.341 ± 0.035
	SGN	**0.394 ± 0.057**	0.393 ± 0.060	0.393 ± 0.059
spruce1722	DBH	**0.286 ± 0.056**	0.268 ± 0.078	0.225 ± 0.060
	DE	**0.433 ± 0.024**	0.424 ± 0.028	0.417 ± 0.022
	HT	**0.347 ± 0.064**	0.334 ± 0.067	0.308 ± 0.041
soy5014	HT	**0.476 ± 0.019**	0.458 ± 0.026	0.453 ± 0.030
	R8	**0.481 ± 0.010**	0.437 ± 0.018	0.456 ± 0.024
	YLD	**0.662 ± 0.007**	0.659 ± 0.010	0.659 ± 0.008
maize5820	DTA	**0.948 ± 0.001**	0.940 ± 0.003	0.942 ± 0.003
	DTS	**0.946 ± 0.004**	0.938 ± 0.005	0.941 ± 0.004
	DTT	**0.948 ± 0.002**	0.939 ± 0.003	0.944 ± 0.003
	EH	**0.941 ± 0.002**	0.921 ± 0.009	0.929 ± 0.001
	ELL	**0.914 ± 0.003**	0.884 ± 0.005	0.893 ± 0.004
	ELW	**0.887 ± 0.007**	0.883 ± 0.008	0.885 ± 0.008
	PH	**0.941 ± 0.003**	0.932 ± 0.004	0.931 ± 0.005
	TBN	**0.927 ± 0.004**	0.905 ± 0.004	0.913 ± 0.006
	TL	**0.924 ± 0.002**	0.902 ± 0.003	0.910 ± 0.004
	CW	**0.852 ± 0.007**	0.815 ± 0.007	0.826 ± 0.013
	ED	**0.840 ± 0.013**	0.552 ± 0.238	0.796 ± 0.094
	EL	**0.831 ± 0.005**	0.803 ± 0.008	0.807 ± 0.010
	ERN	**0.914 ± 0.003**	0.908 ± 0.004	0.908 ± 0.004
	EW	**0.805 ± 0.014**	0.776 ± 0.011	0.782 ± 0.014
	KNPE	**0.847 ± 0.011**	0.841 ± 0.010	0.842 ± 0.012
	KNPR	**0.705 ± 0.016**	0.686 ± 0.018	0.691 ± 0.019
	KWPE	**0.778 ± 0.014**	0.747 ± 0.022	0.756 ± 0.019
	LBT	**0.683 ± 0.017**	0.621 ± 0.106	0.678 ± 0.020

Each value in the cells are means and standard errors of the prediction performance for 5-fold cross-validation. The best results are highlight in **bold**.

## Data Availability

The iADEP scripts are available on github https://github.com/Yechonghang/iADEP (accessed on 24 February 2025). The millet827 dataset is available from https://iagr.genomics.cn/CropGS/#/Datasets?species=Millet (accessed on 24 February 2025) [21]. The spruce1722 dataset and soy5014 dataset are available from https://github.com/FelixHeinrich/GP_with_IFS/tree/main/Datasets (accessed on 24 February 2025) [25]. The maize5820 dataset is available from https://github.com/yingjiexiao/TOP (accessed on 24 February 2025) [27]. The springbarley2463 dataset is available from https://data.mendeley.com/datasets/s3s4ft92wj/1 (accessed on 24 February 2025) [28].

## Supplementary Materials

The following supporting information can be downloaded at: https://www.mdpi.com/article/10.3390/genes16040411/s1. Additional File S1: Supplemental Figures S1–S6 and Supplemental Tables S1–S3. Additional File S2: Supplemental Table S4. Figure S1: Prediction performance of iADEP for traits on four datasets millet827, spruce1722, soy5014, and maize5820; Figure S2: Design of different methods in ablation experiments; Figure S3: The different between transductive learning and inductive learning in 5-fold cross-validation and 100-fold cross-validation; Figure S4: The different output results through CNN; Figure S5: *p*-value of paired sample *t*-test on four datasets using five-fold cross-validation; Figure S6: *p*-value of paired sample *t*-test on springbarley2463 datasets using five-fold cross-validation; Table S1: Overview of the dataset used in this study; Table S2: Hyperparameters used for iADEP in this study; Table S3: Hyperparameters of other methods excluding iADEP in this study; Table S4: The results of feature selection experiment.

## Abbreviations

The following abbreviations are used in this manuscript:

GP	Genomic prediction
GBLUP	Genomic best linear unbiased prediction
ML	Machine learning
RF	Random forest
RR	Ridge regression
SVR	Support vector regression
DL	Deep learning
DeepGS	Deep convolutional neural network
CNN	Convolutional neural networks
DArT	Diversity Array Technology
DNNGP	Deep neural network genomic prediction
SNP	Single nucleotide polymorphisms
GWAS	Genome-wide association study
iADEP	Integrated Additive, Dominant and Epistatic Prediction
MLP	Multilayer perceptrons
LIT	Latent Interaction Testing
XGBoost	eXtreme Gradient Boosting
PCC	Pearson correlation coefficient
iGEP	integrated genomic-enviromic prediction
GradCAM	Gradient-weighted Class Activation Mapping
MAF	Minor allele frequency
RIL	Recombinant lines
GCTA	Genetics complex trait analysis
GREML	Genome-based restricted maximum likelihood
MSE	Mean squared error
TSLL	Top second leaf length
TSLW	Top Second Leaf Width
MSH	Main Stem Height
MSW	Main Stem Width
MSPD	Panicle Diameter of Main Stem
FNL	Fringe Neck Length
MSPL	Panicle Length of Main Stem
MSPW	Main Stem Panicle Weight
PGW	Per Plant Grain Weight
MSSN	Spikelet Number of Main Stem
SGN	Grain Number per Spike
DBG	Diameter at Breast Height
DE	Wood Density
HT	Height
R8	Time to R8 Developmental Stage
YLD	Yield
DTA	Days to Anther
DTS	Days to Silk
DTT	Days to Tassel
EH	Ear Height
ELL	Ear Leaf Length
ELW	Ear Leaf Width
PH	Plant Height
TBN	Tassel Branch Number
TL	Tassel Length
CW	Cob Weight
ED	Ear Diameter
EL	Ear Length
ERN	Ear Row Number
EW	Ear Weight
KNPE	Kernel Number per Ear
KNPR	Kernel Number per Row
KWPE	Kernel Weight per Ear
LBT	Length of Barren Tip
FS	Filtering Speed
EY	Extract Yield
WC	Wort Color
BG	Beta Glucan
WV	Wort Viscosity
