# Supplementary material for: Biological Prior Knowledge-Embedded Deep Neural Network for Plant Genomic Prediction
Source: Genes (Basel). 2025 Mar 31;16(4):411. doi: 10.3390/genes16040411 (PMC12027452; doi:10.3390/genes16040411)
Supplement: Supplementary file 1 [file genes-16-00411-s001.zip › Additional file S1.pdf]

Biological prior knowledge-embedded deep neural network for plant genomic prediction

Chonghang Ye[1], Kai Li[1], Weicheng Sun[1], Yiwei Jiang[1], Ping Zhang[1], Weihan Zhang[2], Yi-Juan Hu[3,], Yuepeng Han[2,*], Li Li[1,4,*]

[1]Agricultural Bioinformatics Key Laboratory of Hubei Province, College of Informatics, Huazhong Agricultural University, Wuhan 430070, China

[2]State Key Laboratory of Plant Diversity and Specialty Crops, Wuhan Botanical Garden, Chinese Academy of Sciences, Hubei Hongshan Laboratory, Wuhan 430074, China

[3]Department of Biostatistics and Bioinformatics, Emory University, Atlanta, GA 30322, USA

[4]Hubei Hongshan Laboratory, Huazhong Agricultural University, Wuhan 430070, China

*Corresponding author: Yuepeng Han (yphan@wbgcas.cn), Li Li (li.li@mail.hzau.edu.cn)

Supporting Information

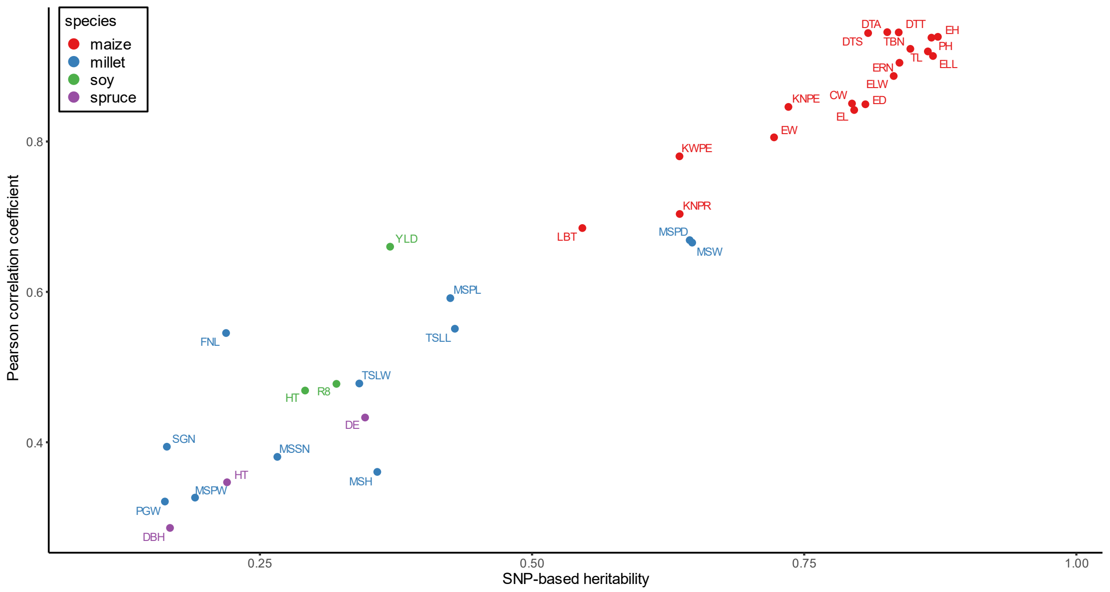

Supplemental Figure S1. Prediction performance of iADEP for traits on four datasets millet827, spruce1722, soy5014, and maize5820.

TSLL, top second leaf length; TSLW, top second leaf width; MSH, main stem height; MSW, main stem width; MSPD, panicle diameter of the main stem; FNL, fringe neck length; MSPL, panicle length of the main stem; MSPW, main stem panicle weight; PGW, per plant grain weight; MSSN, spikelet number of the main stem; SGN, grain number per spike; DBG, diameter at breast height; DE, wood density; HT, height; R8, time to R8 developmental stage; YLD, yield; DTA, days to anther; DTS, days to silk; DTT, days to tassel; EH, ear height; ELL, ear leaf length; ELW, ear leaf width; PH, plant height; TBN, tassel branch number; TL, tassel length; CW, cob weight; ED, ear diameter; EL, ear length; ERN, ear row number; EW, ear weight; KNPE, kernel number per ear; KNPR, kernel number per row; KWPE, kernel weight per ear; LBT, length of barren tip.

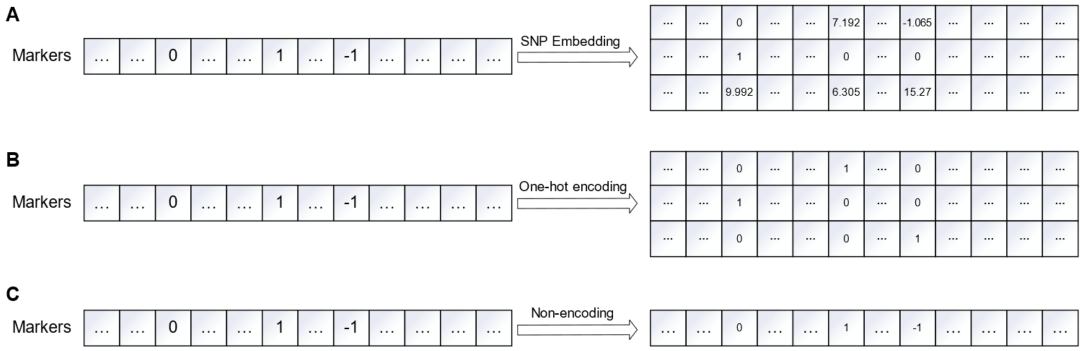

Supplemental Figure S2. Design of different methods in ablation experiments.

(A) SNP embedding**; (B)** One-hot encoding**; (C)** Non-encoding.

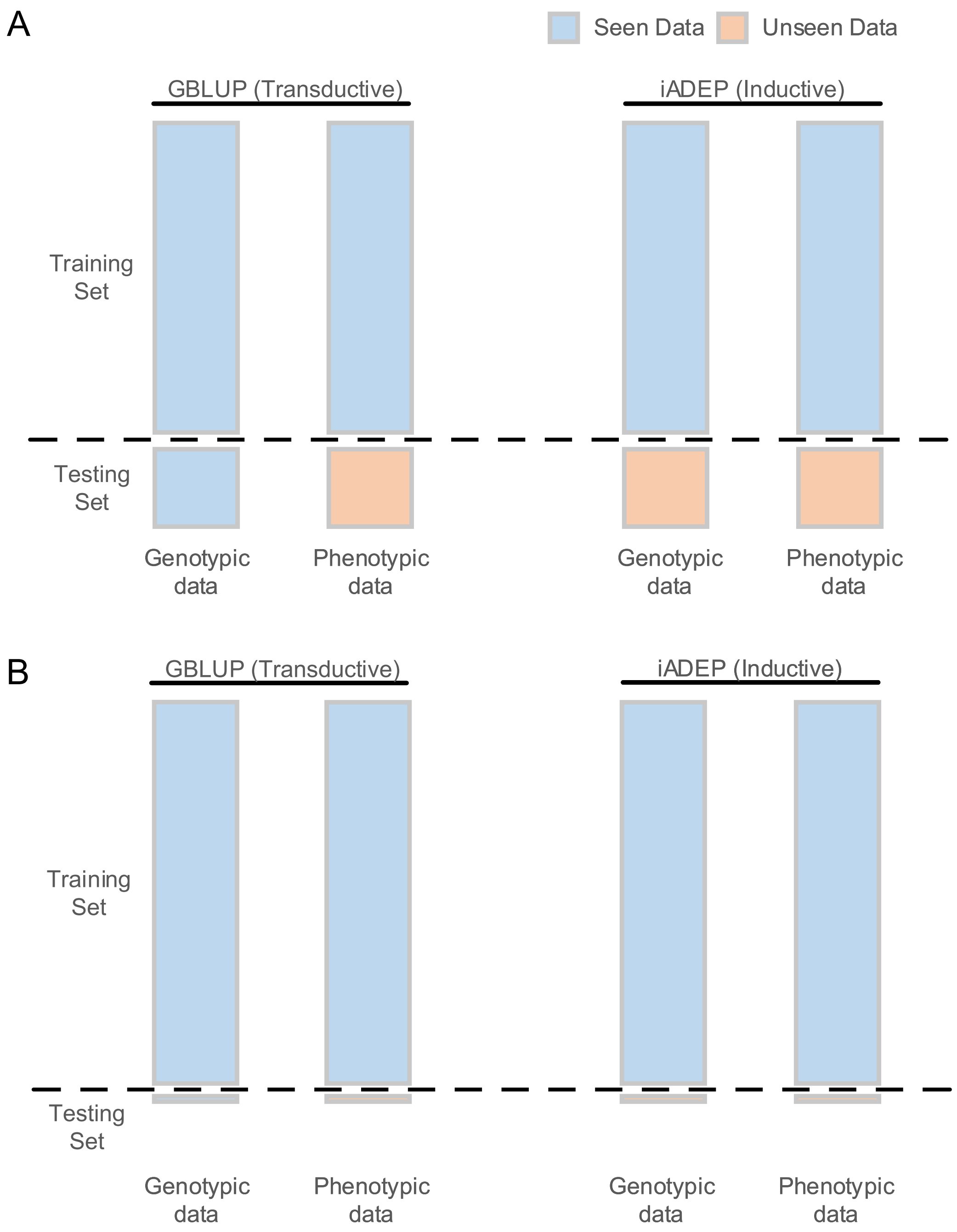

Supplemental Figure S3. The different between transductive learning and inductive learning in 5-fold cross-validation (A) and 100-fold cross-validation (B).

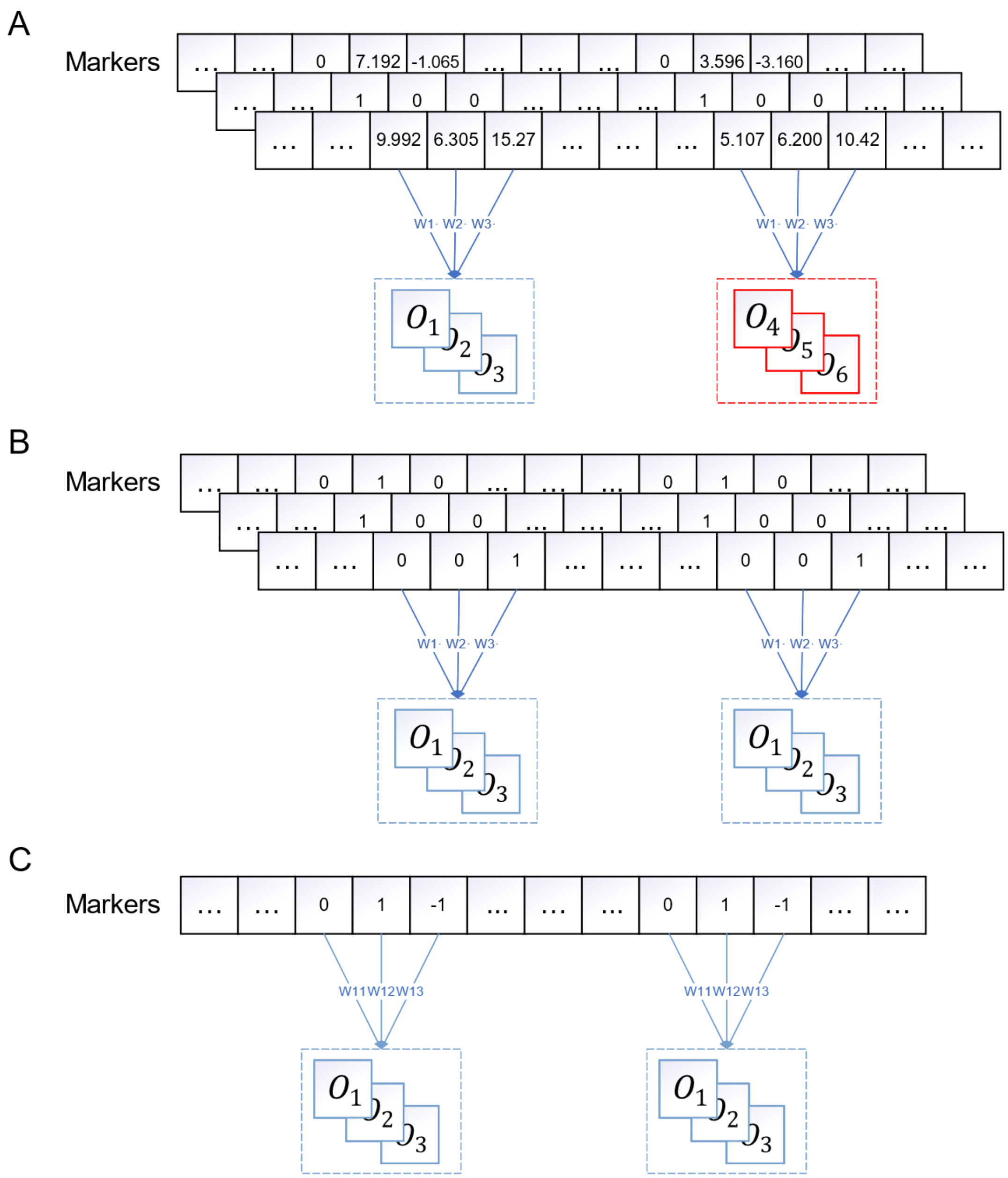

Supplement Figure S4. The different output results through CNN.

(A) SNP embedding; **(B)** One-hot encoding; **(C)** Non-encoding

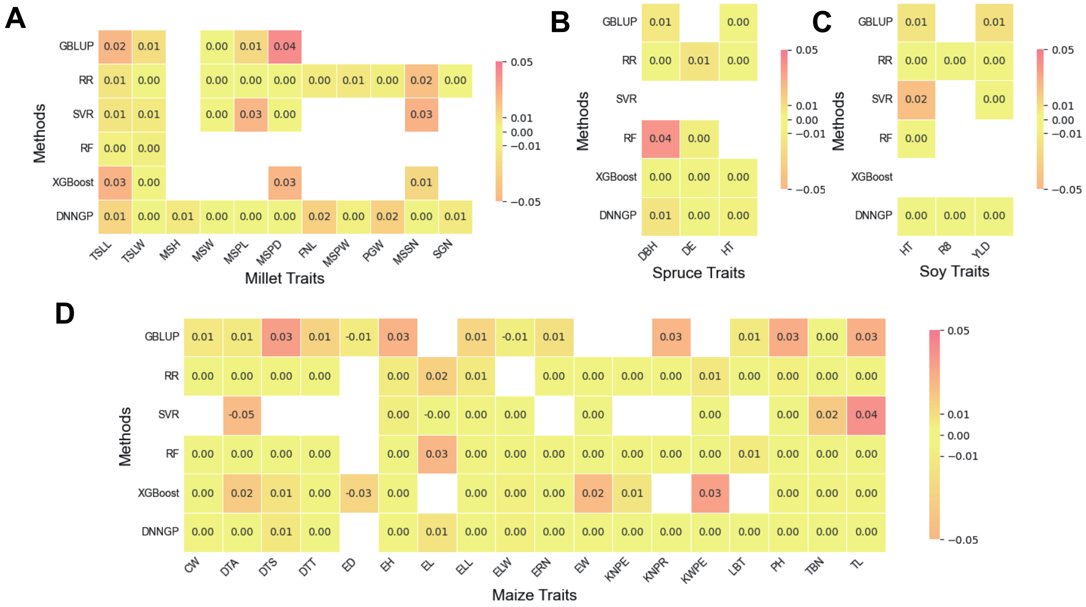

Supplement Figure S5. P-value of paired sample t-test on four datasets using five-fold cross-validation.

(A) millet827 dataset, (B) spruce1722 dataset, (C) soy5014 dataset, (D) maize5820 dataset. Blank entries mean insignificant p-value.

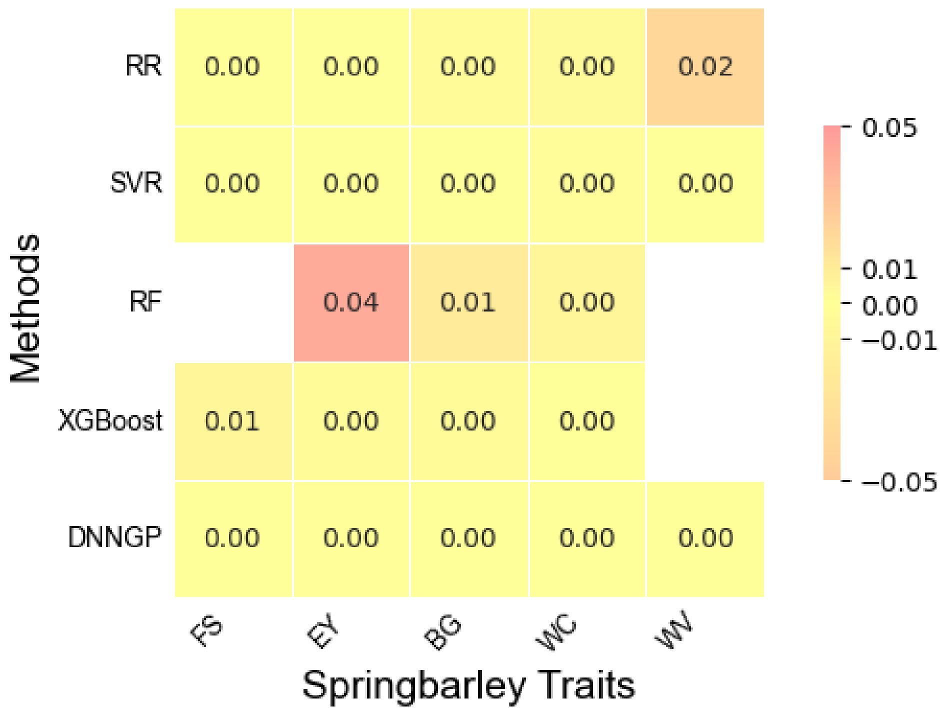

Supplement Figure S6. P-value of paired sample t-test on springbarley2463 datasets using five-fold cross-validation.

Supplemental Table S1. Overview of the dataset used in this study.

Species	Lines (Size)	SNPs	Phenotypes (abbreviation)	
Millet	827 (827)	75780	Top second leaf length (TSLL)	Top second leaf width (TSLW)
			Main stem height (MSH)	Main stem width (MSW)
			Panicle diameter of the main stem (MSPD)	Fringe neck length (FNL)
			Panicle length of the main stem (MSPL)	Per plant grain weight (PGW)
			Main stem panicle weight (MSPW)	Hundred kernel weight (HKW)
			Spikelet number of the main stem (MSSN)	Grain number per spike (SGN)
Spruce	1722 (1722)	5679	Diameter at breast height (DBH)	Wood density (DE)
			Height (HT)	
Soy	5014 (5014)	4234	Height (HT)	Yield (YLD)
			Time to R8 developmental stage (R8)	
Maize	5820 (5820)	156269	Cob weight (CW)	Days to anther (DTA)
			Days to silk (DTS)	Days to tassel (DTT)
			Ear diameter (ED)	Ear height (EH)
			Ear length (EL)	Ear leaf length (ELL)
			Ear leaf width (ELW)	Ear row number (ERN)
			Ear weight (EW)	Kernel number per ear (KNPE)
			Kernel number per row (KNPR)	Kernel weight per ear (KWPE)
			Length of barren tip (LBT)	Plant height (PH)
			Tassel branch number (TBN)	Tassel length (TL)
Spring barley	563 (2463)	3889	Filtering speed (FS)	Extract yield (EY)
			Wort color (WC)	Beta glucan (BG)
			Wort viscosity (WV)	

The data size and SNP data both represent filtered data.

Supplemental Table S2. Hyperparameters used for iADEP in this study.

Dataset	Hyperparameters	TSLL	TSLW	MSH	MSW	MSPL	MSPD	FNL	MSPW	PGW	MSSN	SGN
millet827	learning rate	1e-4	1e-5	1e-4	5e-5	2e-4	1e-5	3e-4	1e-5	5e-5	1e-4	1e-4
	weight decay	1e-6	1e-6	1e-6	1e-6	1e-6	1e-6	1e-6	1e-6	1e-6	1e-6	1e-6
	MLP dropout1	0.7	0.7	0.7	0.7	0.7	0.7	0.7	0.7	0.7	0.7	0.7
	MLPdropout2	0.3	0.3	0.3	0.3	0.3	0.3	0.3	0.3	0.3	0.3	0.3
	batch size	128	128	128	128	128	128	128	128	128	128	128
	first kernel size	21	21	21	21	21	21	21	21	21	21	21
	first kernel stride	11	11	11	11	11	11	11	11	11	11	11
	residual blocks	7	7	7	7	7	7	7	7	7	7	7
	head of attention	8	8	8	8	8	8	8	8	8	8	8
	max epoch	100	100	100	100	100	100	100	100	100	100	100
spruce1722		DBH	DE	HT								
	learning rate	5e-4	5e-4	5e-4								
	weight decay	1e-4	1e-4	1e-4								
	MLP dropout1	0.7	0.7	0.7								
	MLP dropout2	0.3	0.3	0.3								
	batch size	512	512	512								
	first kernel size	7	7	7								
	first kernel stride	3	3	3								
	residual blocks	6	6	6								
	head of attention	16	16	16								
	max epoch	100	100	100								
soy5014		HT	R8	YLD								
	learning rate	3e-4	3e-4	5e-4								
	weight decay	1e-5	1e-5	1e-5								
	MLP dropout1	0.7	0.7	0.7								
	MLP dropout2	0.3	0.3	0.3								
	batch size	1024	1024	1024								
	first kernel size	7	7	7								
	first kernel stride	3	3	3								
	residual blocks	6	6	6								
	head of attention	8	8	8								
	max epoch	100	100	100								
maize5820		CW	DTA	DTS	DTT	ED	EH	EL	ELL	ELW	ERN	EW
	learning rate	1e-4	1e-4	1e-4	1e-4	1e-4	1e-4	1e-4	1e-4	1e-4	1e-4	1e-4
	weight decay	1e-6	1e-6	1e-6	1e-6	1e-6	1e-6	1e-6	1e-6	1e-6	1e-6	1e-6
	MLP dropout1	0.7	0.7	0.7	0.7	0.7	0.7	0.7	0.7	0.7	0.7	0.7
	MLP dropout2	0.3	0.3	0.3	0.3	0.3	0.3	0.3	0.3	0.3	0.3	0.3
	batch size	1024	1024	1024	1024	1024	1024	1024	1024	1024	1024	1024
	first kernel size	21	21	21	21	21	21	21	21	21	21	21
	first kernel stride	11	11	11	11	11	11	11	11	11	11	11
	residual blocks	8	8	8	8	8	8	8	8	8	8	8
	head of attention	8	8	8	8	8	8	8	8	8	8	8
	max epoch	200	200	200	200	200	200	200	200	200	200	200
		KNPE	KNPR	KWPE	LBT	PH	TBN	TL				
	learning rate	1e-4	1e-4	1e-4	1e-4	1e-4	1e-4	1e-4				
	weight decay	1e-6	1e-6	1e-6	1e-6	1e-6	1e-6	1e-6				
	MLP dropout1	0.7	0.7	0.7	0.7	0.7	0.7	0.7				
	MLP dropout2	0.3	0.3	0.3	0.3	0.3	0.3	0.3				

	batch size	1024	1024	1024	1024	1024	1024	1024
	first kernel size	21	21	21	21	21	21	21
	first kernel stride	11	11	11	11	11	11	11
	residual blocks	8	8	8	8	8	8	8
	head of attention	8	8	8	8	8	8	8
	max epoch	200	200	200	200	200	200	200
Springbarley 2463		FS	EY	BG	WC	WV		
	learning rate	5e-5	5e-5	5e-5	5e-5	5e-5		
	weight decay	1e-2	1e-2	1e-2	1e-2	1e-2		
	MLP dropout1	0.7	0.7	0.7	0.7	0.7		
	MLP dropout2	0.3	0.3	0.3	0.3	0.3		
	batch size	512	512	512	512	512		
	first kernel size	7	7	7	7	7		
	first kernel stride	3	3	3	3	3		
	residual blocks	5	5	5	5	5		
	head of attention	8	8	8	8	8		
	max epoch	150	150	150	150	150		

TSLL, top second leaf length; TSLW, top second leaf width; MSH, main stem height; MSW, main stem width; MSPD, panicle diameter of the main stem; FNL, fringe neck length; MSPL, panicle length of the main stem; MSPW, main stem panicle weight; PGW, per plant grain weight; MSSN, spikelet number of the main stem; SGN, grain number per spike; DBG, diameter at breast height; DE, wood density; HT, height; R8, time to R8 developmental stage; YLD, yield; DTA, days to anther; DTS, days to silk; DTT, days to tassel; EH, ear height; ELL, ear leaf length; ELW, ear leaf width; PH, plant height; TBN, tassel branch number; TL, tassel length; CW, cob weight; ED, ear diameter; EL, ear length; ERN, ear row number; EW, ear weight; KNPE, kernel number per ear; KNPR, kernel number per row; KWPE, kernel weight per ear; LBT, length of barren tip; FS, filtering speed; EY, extract yield; WC, wort color; BG, beta glucan; WV, wort viscosity.

Supplemental Table S3. Hyperparameters of other methods excluding iADEP in this study.

Hyperparameters of DNNGP and RF

methods	learning rate	drouput1	drouput2	batch size	max epoch
DNNGP	1e-3	0.5	0.3	28	300
RF	Default hyperparameters in Python package "scikit-learn"				

Hyperparameters of RR, SVR, and XGBoost

Datasets	Methods	Hyperparameters	Traits					
Millet827			TSLL	TSLW	MSH	MSW	MSPL	MSPD
	RR	alpha	100	100	100	100	100	100
	SVR	kernel	linear	poly	rbf	rbf	poly	linear
		C	1	0.1	100	10	10	1
	XGBoost	n_estimators	50	50	50	50	50	50
		max_depths	4	8	8	6	7	4
		gamma	01	0	0	0	0.3	0.4
		subsample	0.7	1	1	1	1	1
		reg_alpha	100	10	100	1	100	100
		learning_rate	0.1	0.1	0.1	0.1	0.1	0.1
			FNL	MSPW	PGW	MSSN	SGN	
	RR	alpha	100	100	100	100	100	
	SVR	kernel	rbf	rbf	rbf	sigmoid	sigmoid	
		C	10	10	10	1	100	
	XGBoost	n_estimators	50	50	50	50	50	
		max_depths	8	5	5	4	6	
		gamma	0.3	0.3	0.3	0	0.4	
		subsample	1	1	1	1	1	
		reg_alpha	100	0.01	100	100	0	
		learning_rate	0.1	0.05	0.05	0.05	0.2	
Spruce1722			DBH	DE	HT			
	RR	alpha	100	100	100			
	SVR	kernel	rbf	poly	rbf			
		C	1	1	1			
	XGBoost	n_estimators	50	100	100			
		max_depths	4	4	5			
		gamma	0.3	0.2	0			
		subsample	1	1	1			
		reg_alpha	0	0.1	0.1			
		learning_rate	0.05	0.05	0.05			
Soy5014			HT	R8	YLD			
	RR	alpha	100	100	100			
	SVR	kernel	rbf	rbf	rbf			
		C	1	1	1			
	XGBoost	n_estimators	50	50	50			
		max_depths	4	4	4			
		gamma	0	0	0			
		subsample	0.9	1	0.9			
		reg_alpha	10	10	10			
		learning_rate	0.1	0.1	0.2			

			CW	DTA	DTS	DTT	ED	EH
Maize5820	RR	alpha	100	100	100	100	100	100
	SVR	kernel	rbf	rbf	rbf	rbf	rbf	linear
		C	10	1	1	1	0.1	0.01
	XGBoost	n_estimators	50	50	50	50	50	50
		max_depths	4	4	4	4	4	4
		gamma	0.3	0	0.1	0.3	0	0
		subsample	1	1	1	1	1	1
		reg_alpha	100	10	10	10	1	100
		learning_rate	0.2	0.2	0.2	0.2	0.2	0.2
			EL	ELL	ELW	ERN	EW	KNPE
	RR	alpha	100	100	100	100	100	100
	SVR	kernel	rbf	rbf	rbf	rbf	linear	linear
		C	1	10	1	1	0.01	0.01
	XGBoost	n_estimators	50	50	50	50	50	50
		max_depths	4	4	4	4	4	5
		gamma	0.4	0	0.3	0	0	0
		subsample	1	1	1	1	1	1
		reg_alpha	10	100	1	10	100	1
		learning_rate	0.2	0.2	0.2	0.2	0.2	0.2
			KNPR	KWPE	LBT	PH	TBN	TL
	RR	alpha	100	100	100	100	100	100
	SVR	kernel	rbf	linear	rbf	linear	linear	linear
		C	1	0.1	1	0.01	1	1
	XGBoost	n_estimators	50	50	50	50	50	50
		max_depths	4	4	4	4	4	5
		gamma	0.1	0	0	0	0.1	0.1
		subsample	0.9	1	1	1	0.9	1
		reg_alpha	10	100	10	100	1	10
		learning_rate	0.2	0.3	0.2	0.3	0.2	0.2

DNNGP, deep neural network for genomic prediction; RR, ridge regression; SVR, support vector regression; RF, random forest; XGBoost, extreme gradient boosting. TSLL, top second leaf length; TSLW, top second leaf width; MSH, main stem height; MSW, main stem width; MSPD, panicle diameter of the main stem; FNL, fringe neck length; MSPL, panicle length of the main stem; MSPW, main stem panicle weight; PGW, per plant grain weight; MSSN, spikelet number of the main stem; SGN, grain number per spike; DBG, diameter at breast height; DE, wood density; HT, height; R8, time to R8 developmental stage; YLD, yield; DTA, days to anther; DTS, days to silk; DTT, days to tassel; EH, ear height; ELL, ear leaf length; ELW, ear leaf width; PH, plant height; TBN, tassel branch number; TL, tassel length; CW, cob weight; ED, ear diameter; EL, ear length; ERN, ear row number; EW, ear weight; KNPE, kernel number per ear; KNPR, kernel number per row; KWPE, kernel weight per ear; LBT, length of

barren tip; FS, filtering speed; EY, extract yield; WC, wort color; BG, beta glucan; WV, wort viscosity.
